# A bathypelagic ostracod *Conchoecissa nigromaculatus* sp. nov. (Myodocopa, Halocyprididae) from the South China Sea

**DOI:** 10.7717/peerj.5557

**Published:** 2018-09-07

**Authors:** Peng Xiang, Yu Wang, Ruixiang Chen, Liyuan Zhao, Chunguang Wang, Mao Lin

**Affiliations:** 1 School of Life Science and Technology, Tongji University, Shanghai, China; 2 Laboratory of Marine Biology and Ecology, Third Institute of Oceanography, SOA, Xiamen, China; 3 Collaborative Innovation Center of Deep Sea Biology, Hangzhou, China

**Keywords:** Taxonomy, Ostracoda, Bathypelagic, *Conchoecissa nigromaculatus* sp. nov., South China Sea

## Abstract

Pelagic ostracods are one of the main groups of zooplankton and are abundant in marine ecosystems worldwide. The record of marine planktonic ostracod species in the central and southern part of the South China Sea accounts over for one-third of the total recorded marine planktonic ostracods in seas around China. In this study, we examined and compared the specimens from a recent cruise in this region that appeared to be different from previously described species of genus *Conchoecissa*, and then confirmed them as a new bathypelagic species *Conchoecissa nigromaculatus*. These specimens clearly differed from the other species of genus *Conchoecissa* with differences observed in the size, carapace, locations of glands, mandible, maxilla, sixth limb, and furca. In this species, mandibular coxal endite has no ventral finger process, maxilla has prominently large endites and has only two claws on the tip, the sixth limb has very simple endites, and this species has distinctive features not previously observed in the tribe Conchoeciini before. It is therefore necessary to emend the diagnosis of this group.

## Introduction

Marine zooplankton form a main link between primary producers and higher trophic levels in the food web of marine ecosystems, and are a key element in the fluxes of carbon through the ocean surface to bottom. Pelagic ostracods occur throughout oceanic water columns and are often the second- or third-most abundant taxon in mesoplankton samples particularly at sub-thermocline depth. Despite their abundance, the importance of their role in influencing carbon fluxes are often overlooked because they are generally considered to be difficult to identify. The classification of pelagic ostracods is based on morphological features that are highly diverse. The group may not be monophyletic, and phylogenetics of the species remains ambiguous (Fortey & Richard, 1997; Yamaguchi & Endo, 2003). In last two decades, the taxonomy and ecology of marine ostracods has been extensively revised and improved. The subclass Ostracoda has been emended to class Ostracoda Latreille, 1802, emend. Martin & Davis, 2001, and many new taxa have been reported and described (Harrison-Nelson & Kornicker, 2000; Chavtur, 2003; Lum et al., 2008; Karanovic, 2010; Chavtur & Angel, 2011; Xiang et al., 2017a).

The tribe Conchoeciini (Chavtur & Angel, 2011), is included in the family Halocyprididae (Dana, 1853), the most speciose family of order Halocyprida (Dana, 1853), and the second-most speciose family of subclass Myodocopa (Sars, 1866; Chavtur, 2003; Brandão et al., 2018). Tribe Conchoeciini currently contains 21 genera including the genus *Conchoecissa* (Claus, 1890), which was originally erected as a monospecific genus including just *C. imbricata* (Brady, 1880). However, Müller (1906) confusingly re-classified many of Claus’s halocyprid genera as *Conchoecia* species, which he subdivided into ‘species-groups’. *Conchoecissa imbricata* became part of his Imbricata-Group (Müller, 1906) in which he included four new species: *C. plinthina* (Müller, 1906), *C. symmetrica* (Müller, 1906), *C. ametra* (Müller, 1906) and *C. squamosa* (Müller, 1906). Poulsen (1973) began the process of re-establishing Claus’s genera, and placed all Müller’s Imbricata-Group species back in *Conchoecissa*. Since then, no further species have been added to the genus.

Far away from many of the pressures of anthropogenic influence, the South China Sea supports a rich marine biodiversity, including a large number of coral reef communities, yet for most marine taxa is relatively understudied (Kasim, 2000; Li et al., 2005; Chen, 2011; Liu, 2013). A total of 81 marine planktonic ostracod species have been recorded in the central and southern part of South China Sea (detailed species list is given in Table S1), which accounts for just over one-third of the total recorded of marine planktonic ostracods in seas around China (=242 species, Chen, 2012). Most of these South China Sea species were recorded during six cruises of the “Comprehensive Investigations on Environmental Resources in the Central Part of the South China Sea” project from April 1983 to January 1985 by the zooplankton research group in Third Institute of Oceanography, SOA (Chen, Cai & Lin, 1988), as well as new species discovered recently (Yin, Chen & Li, 2014; Xiang et al., 2017b; Yin, Li & Tan, 2017). The collections of ostracods from a recent cruise in the South China Sea included specimens that appeared to be different from previously described species of genus *Conchoecissa*. Here we examined and compared these specimens to assess whether these represent a new species.

## Materials and Methods

The collections that included the novel species were made during a cruise in the southern South China Sea from December 2013 to January 2014, between latitudes 7°N to 12°N and longitudes 110°E to 115°E.

All zooplankton samples were collected using a Hydro-Bios Multinet system (Type Midi, mouth 0.25 m^2^, mesh-size aperture 200 µm; HydroBios Inc., Kiel, Germany) by vertical and stratified hauls from bottom to surface. Specimens were photographed live using a Canon 7D camera fitted with an EF100 mm USM Micro lens (Canon Inc., Tokyo, Japan), before being preserved in 5% buffered formaldehyde for preservation.

The specimens were dissected under a zoom-stereomicroscope system (Discovery V20; Zeiss, Oberkochen, Germany) and mounted on permanent slides with CMC-9AF mounting medium (Masters Company Inc., Wood Dale, IL, USA). Observations, measurements, micrographs and drawings were made following the methodology of Chavtur & Angel (2011) using a transmitted-light binocular microscope combined with a differential interference contrast system (Axio Imager Z2) and an AxioVision Image-Pro software (Carl Zeiss Inc., Oberkochen, Germany). All drawings were made of preserved specimens using a camera Lucida and drawing apparatus. Figures were finally prepared with Adobe Photoshop CS6 software (Adobe Inc., San Jose, CA, USA). Specimens/appendages have been archived in the Marine Biological Sample Museum, the Third Institute of Oceanography, SOA (Xiamen, China), under the collection Nos. TIO-OHHZe-01 and TIO-OHHZe-02.

### Nomenclatural acts

The electronic version of this article in Portable Document Format (PDF) will represent a published work according to the International Commission on Zoological Nomenclature (ICZN), and hence the new names contained in the electronic version are effectively published under that Code from the electronic edition alone. This published work and the nomenclatural acts it contains have been registered in ZooBank, the online registration system for the ICZN. The ZooBank LSIDs (Life Science Identifiers) can be resolved and the associated information viewed through any standard web browser by appending the LSID to the prefix http://zoobank.org/. The LSID for this publication is: urn:lsid:zoobank.org:pub:D5DFB0CC-37C5-4A75-BB4B-5A4FF9A81747. The online version of this work is archived and available from the following digital repositories: PeerJ, PubMed Central and CLOCKSS.

## Results

### Systematic account

**Table:** 

**Figure 1:**
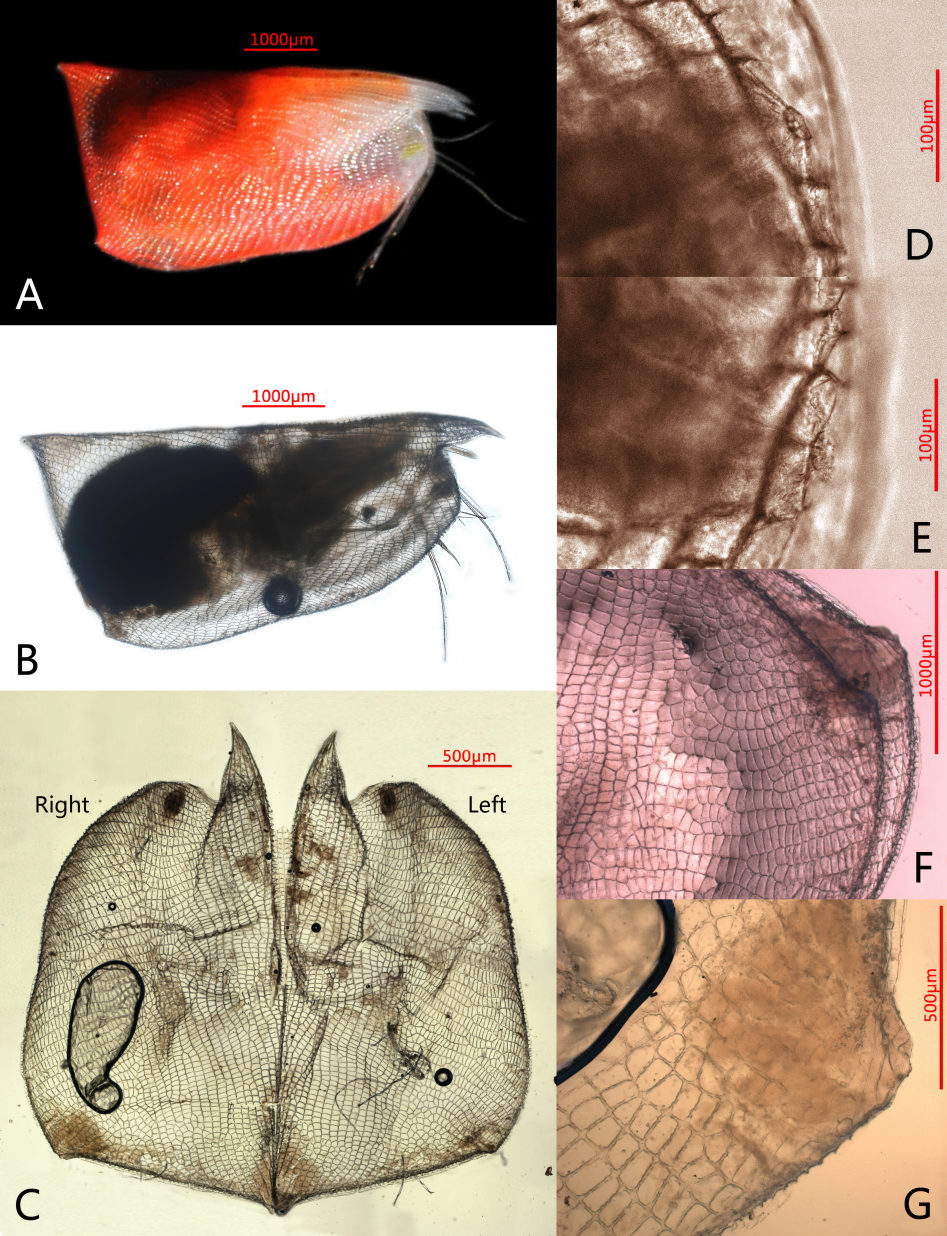
Photomicrographs of *Conchoecissa nigromaculatus* sp. nov., ♀. (A) Right side of living specimen, lateral view. (B) Right valve, lateral view. (C) Valves, medial view. (D) Antero-ventral margin of left valve, lateral view. (E) Antero-ventral margin of right valve, lateral view. (F) Both postero-ventral corners of valves, lateral view. (G) Postero-ventral corner of right valve, medial view.

**Figure 2:**
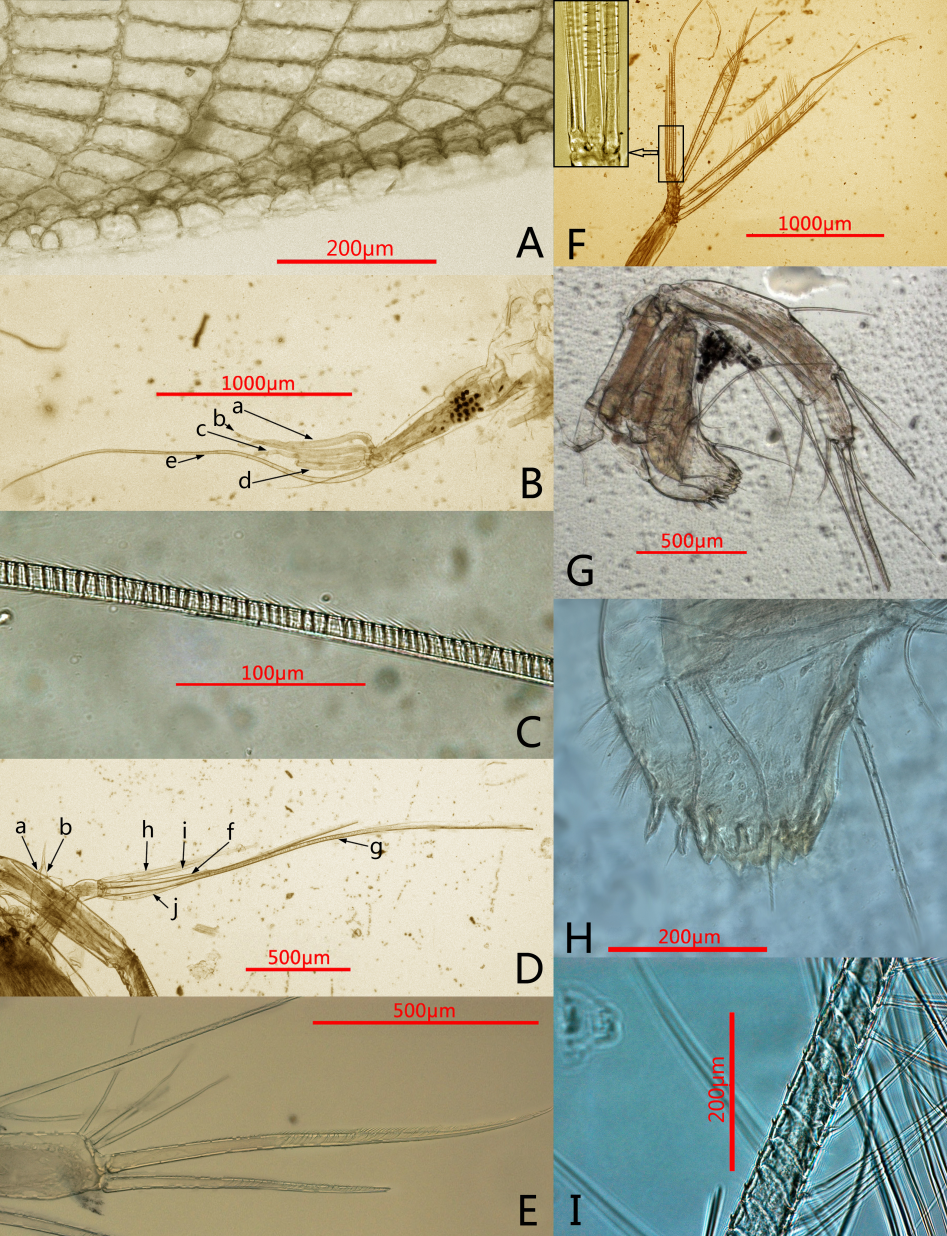
Photomicrographs of *Conchoecissa nigromaculatus* sp. nov., ♀. (A) Ventral margin of valves, lateral view. (B) First antenna, lateral view. (C) E-seta of first antenna, lateral view. (D) Exopod of second antenna, medial view. (E) Tip of mandible, lateral view. (F) Endopod of second antenna, medial view; upper left shows the detail of segment 9 and setae. (G) Mandible, medial view. (H) Mandible, toothed edge of basale, medial view. (I) Detail of plumose setae of exopod of 2nd antenna.

**Figure 3:**
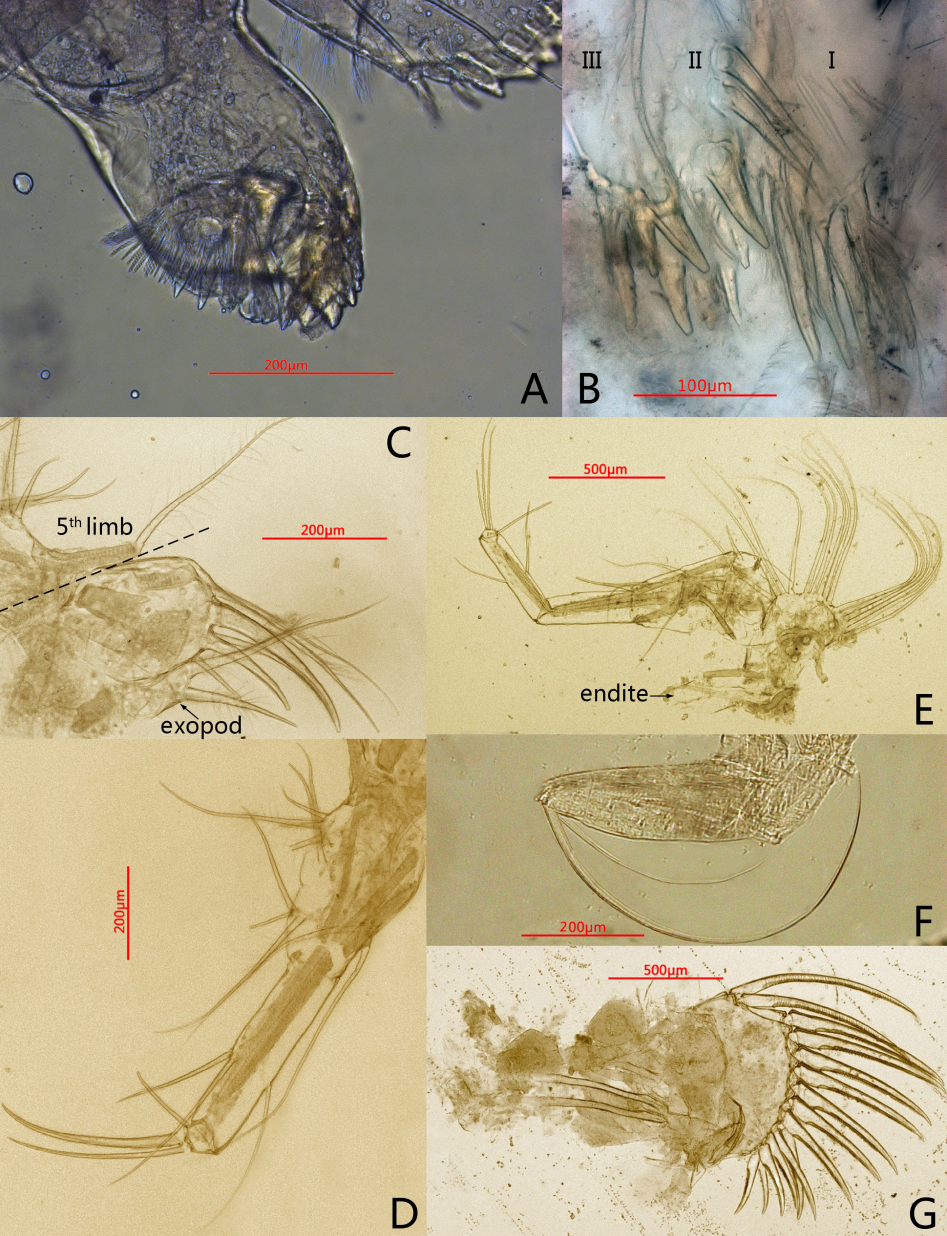
Photomicrographs of *Conchoecissa nigromaculatus* sp. nov., ♀. (A) Mandible, coxal endite, medial view. (B) Maxilla, endites, medial view. (C) Endopod of maxilla, lateral view. (D) Fifth limb, lateral view. (E) Sixth limb, lateral view (F) Seventh limb, medial view. (G) Furcal lamellae, lateral view.

**Figure 4:**
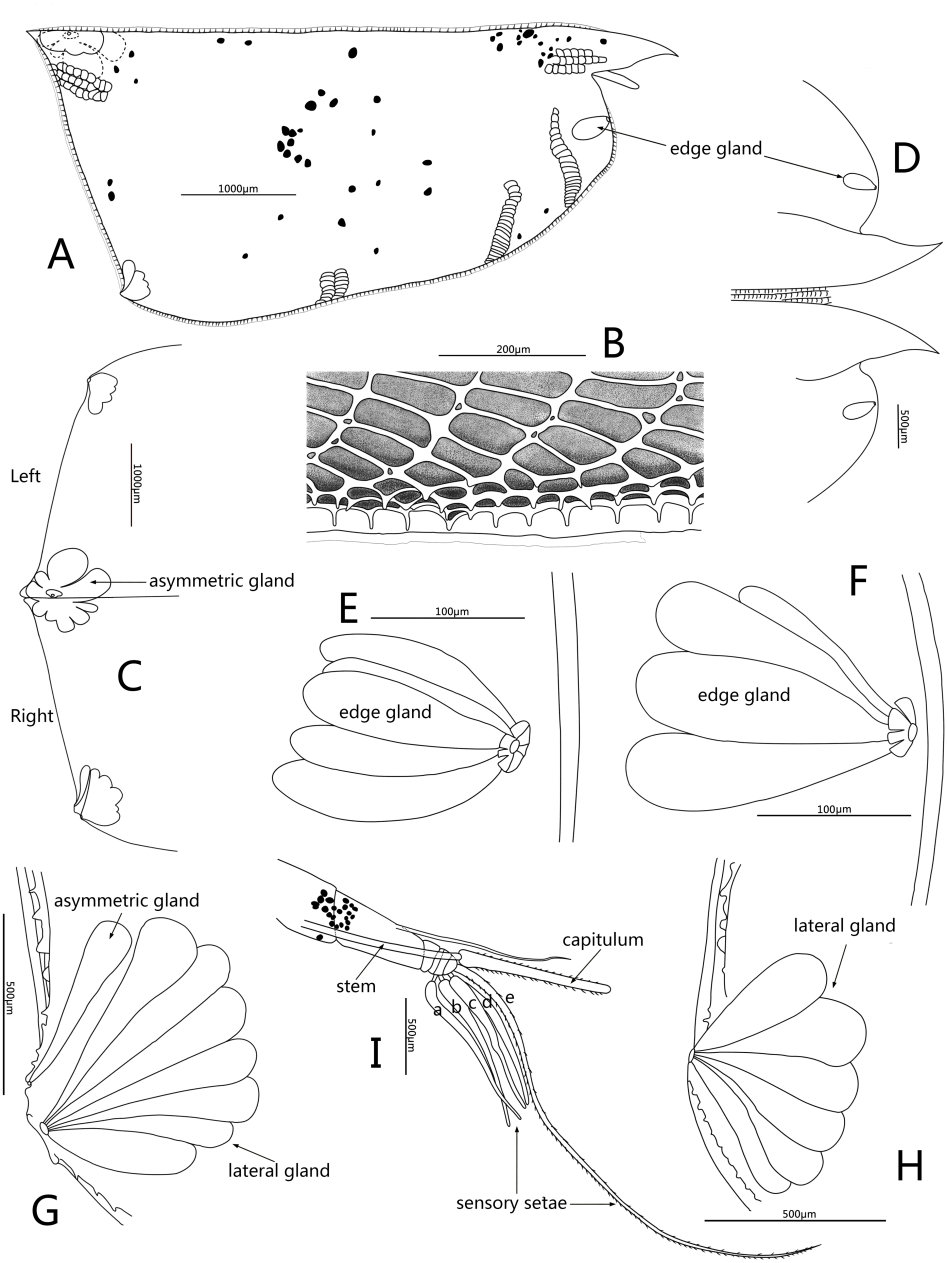
Line drawings of *Conchoecissa nigromaculatus* sp. nov., ♀. (A) Right valve, lateral view. (B) Ventral margin of valves, lateral view. (C) Posterior part of valves, lateral view. (D) Anterior part of valves, lateral view. (E) Antero-ventral margin of left valve, lateral view. (F) Antero-ventral margin of right valve, medial view. (G) Postero-ventral corner of right valve, lateral view. (H) Postero-ventral corner of left valve, medial view. (I) First antenna and frontal organ, lateral view.

**Figure 5:**
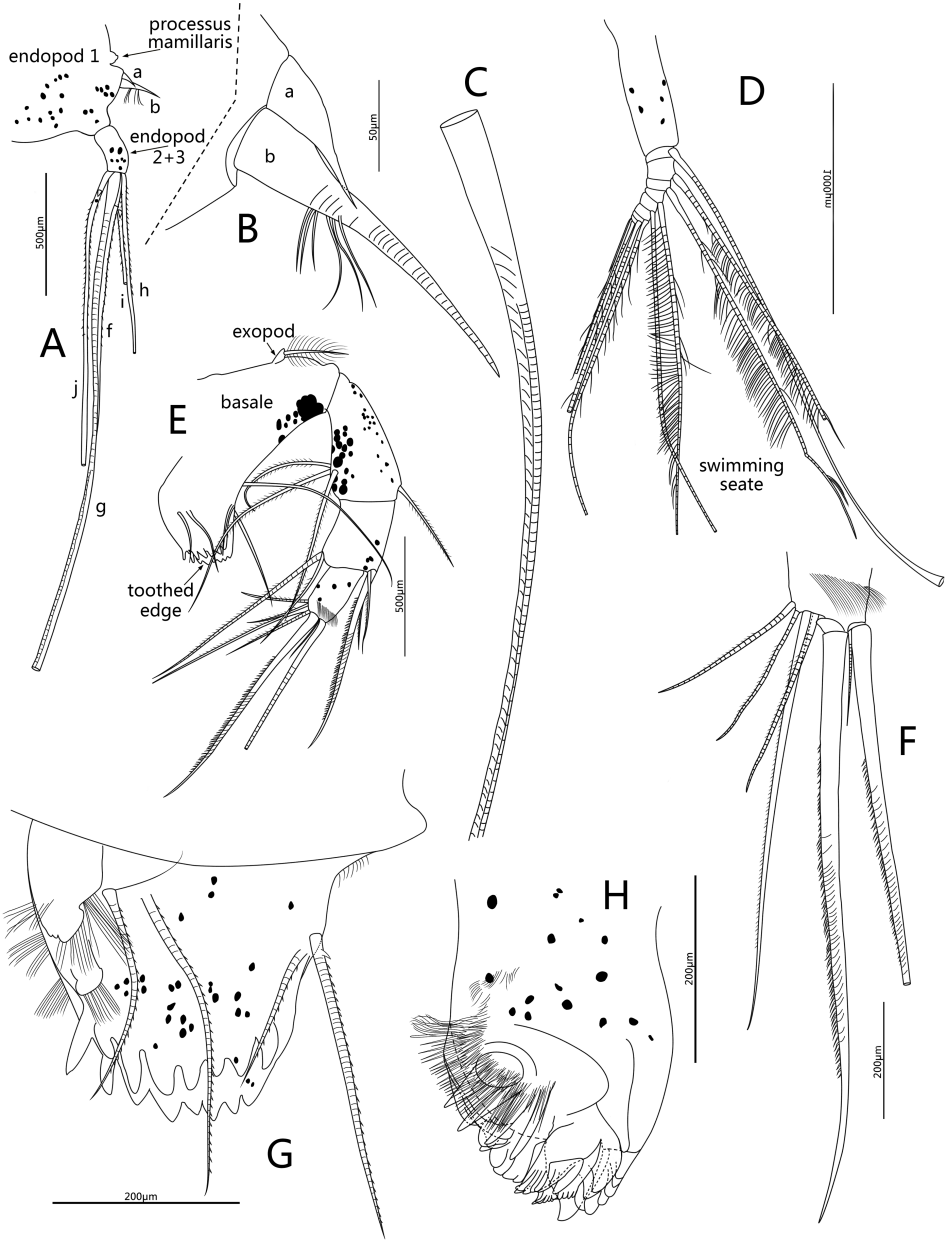
Line drawings of *Conchoecissa nigromaculatus* sp. nov., ♀. (A) Second antenna, medial view (B) A- and b-setae of endopod of second antenna, medial view. (C) H-seta of second antenna. (D) Exopod of second antenna, medial view. (E) Mandible, medial view. (F) Tip of mandibular endopod, medial view. (G) Mandible, toothed edge of basale, medial view. (H) Mandible, coxal endite, medial view.

**Figure 6:**
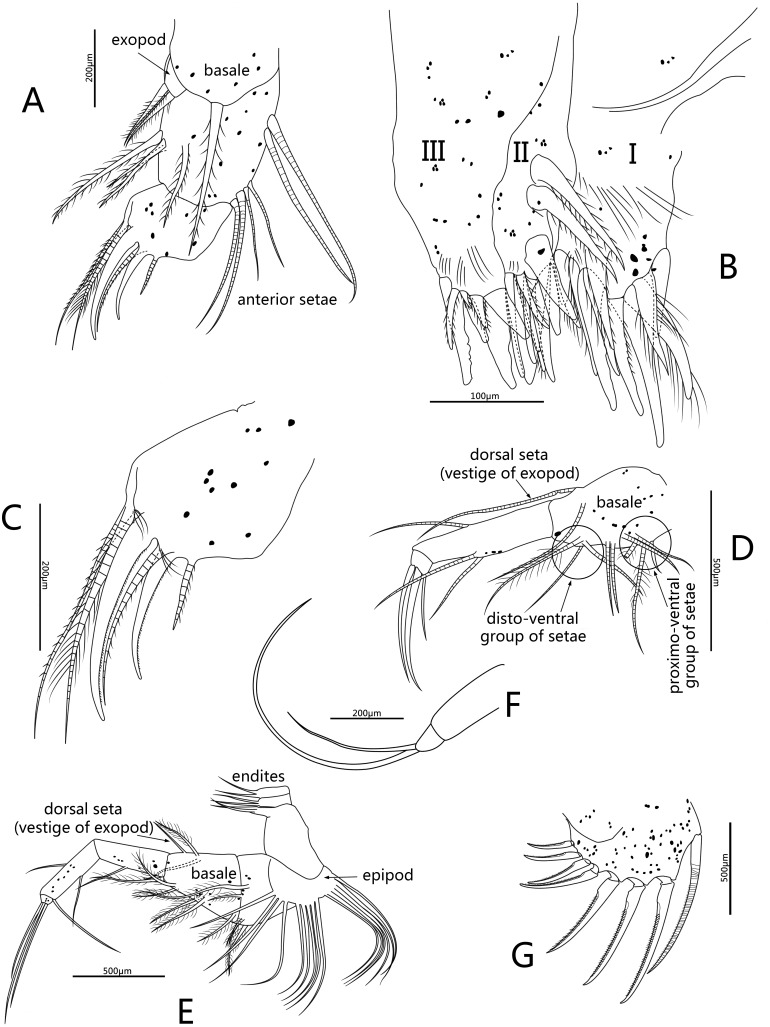
Line drawings of *Conchoecissa nigromaculatus* sp. nov., ♀. (A) Maxilla, lateral view. (B) Endites of maxilla, lateral view. (C) Tip of maxilla, lateral view. (D) Fifth limb, lateral view. (E) Sixth limb, lateral view. (F) Seventh limb, lateral view. (G) Furcal lamella, lateral view.

**Order** Halocypridina Dana, 1853
**Family** Halocyprididae Dana, 1853
**Subfamily** Conchoeciinae G.W. Müller, 1912
**Tribe** Conchoeciini Chavtur & Angel, 2011
**Genus** *Conchoecissa* Claus, 1890
**Species** ***Conchoecissa nigromaculatus*** **Xiang, Wang** & **Chen sp. nov.**
urn:lsid:zoobank.org:act:AE68FC48-CBD1-4090-84AE-27913DB59BA5
Figs. 1–6

**Etymology. “***nigromaculatus*” derived from Latin expression of “dark spots”, indicates this species has dark spots covering the carapace and limbs.

**Holotype.** No. TIO-OHHZe-01, adult female, length 6.1 mm, height 2.7 mm. Type locality: 113°53.6′E, 7°6.46′N, Sounding 1,965 m (Station CS-012) in the South China Sea at a depth of 1,000–1,200 m, on 2nd January 2014.

**Paratype.** No. TIO-OHHZe-02, adult female, length 5.8 mm, height 2.6 mm, was collected at Station CS-015 (112°32.3′E, 7°28.9′N, Sounding 2,086 m) in the South China Sea, at a depth of 1,000–1,500 m on 4th January 2014. Paratype dissected on slides, deposited with holotype.

**Distribution.** In bathyal waters of the southern South China Sea.

**Diagnosis.** Large size. Surface with small dark spots covering. Height about 44% of length, breadth about 32.8 % of length. Carapace subtrapezoidal in lateral view with polygonal pits, without setae; rostrum elongated anteriorly; antero-ventral margins curved; dorsal and posterior margin straight; ventral margin with slightly concavity; postero-ventral corner slightly developed; postero-dorsal corners acutely angled. Postero-dorsal margin of left valve with asymmetric gland near postero-dorsal corner; antero-ventral margin of each valve circular arced with one edge gland; postero-ventral corner of each valve developed into small tubercle. Asymmetric gland on right valve opening on tubercle with one alongside a lateral gland. A lateral gland also opening on protuberance on right valve. On endopod of second antenna, c-, d- and e-setae absent. Mandibular coxal endite without ventral finger process. Maxilla with three large endites. Endopod 2 of sixth limb with mid-dorsal, mid-ventral and disto-ventral setae; endites very simple. Furcal lamellae without unpaired seta.

### Description

**Color** (Fig. 1A): Living specimens red, like most species in this genus, glands on antero-ventral margin of valves yellow. Carapace and appendages with small dark spots on surfaces.

**Carapace** (Figs. 1A–1G, 2A, 4A–4H): Carapace bare, subtrapezoidal in lateral view with polygonal pits covering, thin and transparent edges. Rostra long, subequal, slightly curved and with sharp tips. Dorsal and posterior margin straight. Antero-ventral margin rounded, and ventral margin slightly concave. Both postero-ventral corners developed into small tubercles; right one a bit bigger. Both postero-dorsal corners cutely angled, left corner slightly bigger. Carapace with six groups of glands: a large group of edge gland opening on antero-ventral margin of each valve; a large lateral gland opening on each of tubercles at postero-ventral corner; postero-ventral gland opening alongside large asymmetric gland on right valve, whereas left asymmetric gland opening at usual position for Conchoeciini on dorsal margin just anterior to back end of hinge between two valves. Length 5.8–6.1 mm, height 2.6–2.7 mm, breadth 1.9–2.0 mm, Hence maximum height about 44.5% of length, breadth about 32.8% of length.

**Frontal organ** (Figs. 1B, 4A, 4I): Stem very long, straight and thin. Capitulum spinose, straight and clavate, with slightly thickened base.

**First antenna** (Figs. 2B–2C, 4I): Antenna uniramous with six segments. Segment 1 bare. Segment 2 twice as long as segment 1, and with one long dorsal plumose seta. Segments 3–6 quite short. Segments 3 and 4 bare. Segment 5 with two ventral sensory setae: a- and b-setae. Segment 6 with two equilong ventral sensory setae and one distal sensory seta: c- to e-setae. A- to d-setae subequal, bare and a third length of e-seta. E-seta with short spines along its trailing edge.

**Second antenna** (Figs. 2D, 2F, 2I, 5A–5D): Antenna biramous. Protopodite extremely large with powerful muscles. Endopod without c-, d-, e-setae. Endopod with three segments. Segment 1 with inflated base, small processus mamillaris, a-seta short curved and half length of b-seta also curved with two clusters of long cilia on its mid-ventral margin. Segments 2 and 3 fused with five setae: h- and i-setae thin and spinous; f- and g-setae three times length of h-seta, with flat distal half; j-seta two times length of h-seta; all five setae with bare distal half. Exopod with nine segments. Segment 1 long and bare, segments 2 to 8 with one long plumose swimming seta with tiny spines, respectively; seta 1 with flat distal half part. Segment 9 with one plumose seta, one long acerose seta and one very small acerose seta, arranged from long to short sequence.

**Mandible** (Figs. 2E, 2G–2H, 3A, 5E–5H): Basale large. Exopod tiny thumb shaped process with a single long plumose seta on tip. Endopod with three segments. Segment 1 with one disto-dorsal and three ventral long spinose setae. Segment 2 with two short setae and one long claw with disto-half ventral short spines, on disto-dorsal margin; one quite long and one short setae with disto-half ventral short spines, on disto-ventral margin. Segment 3 with one row of dorsal cilia on distal part and seven setae on tip: four short disto-ventral setae, two long knife-shaped claws with several disto-half short ventral spines, and one small bare seta between claws. Toothed edge of basale with four long slim spinose setae on proximal margin, three clusters of short setae on medio-dorsal side, short medial cilia on proximo-ventral margin, and two lists of teeth, eight on distal list and seven on proximal list. Coxal endite constituting by two parts: medio-ventral part with one big triangular tooth, four comb teeth and three little teeth; medio-dorsal part with four long teeth, four plate teeth, one comb tooth, one little tooth, one cluster of short soft cilia, one cluster of long soft cilia, one row of long sclerotic outer spines and one row of short sclerotic inner spines. Coxal endite without ventral finger process.

**Maxilla** (Figs. 3B–3C, 6A–6C): Maxilla with three large endites. Each endite with a row of medial cilia. Endite I with about seven spinose setae and five papillae. Endite II with two large medio-anterior plumose setae, two plumose distal setae and about seven long papillae. Endite III with about three short plumose setae, two short and two long papillae. Exopod with two spinose setae on tip. Endopod 1 with two long anterior setae, four antero-distal setae, one long spinose lateral seta, three posterior spinose setae, one disto-posterior plumose seta and one spinose medial seta. Endopod 2 with six setae arranged from long to short sequence: seta 1with dorsal spines, seta 2 plumose, setae 4 and 6 with ventral spines, setae 3 and 5 claw-shaped with several ventral spines; setae 2 and 4 with one cluster of long cilia on base.

**Fifth limb** (Figs. 3D, 6D): Endopod with three segments. Segment 1 large with four groups of setae: proximo-ventral group with two long plumose and three short setae; mid-ventral group with two bare setae; disto-ventral group with one long spinose and two shorter setae; disto-dorsal group with two longer setae. Segment 2 long and thin with two ventral and one dorsal setae. Segment 3 short and small with three bare and long acerose setae on tip. Epipod with one small bare inner seta and about fourteen long flexible setae.

**Sixth limb** (Figs. 3E, 6E): Coxale broad and short with one pair of short disto-ventral setae, one long disto-ventral seta, one long and one short disto-medial setae. Basale large and long, with one pair of long mid-dorsal setae, one disto-dorsal seta (vestige of exopod), one proximal long seta, one pair of long mid-ventral setae, one pair of disto-ventral setae and one long seta on disto-ventral edge. Endopod with three segments. Segment 1 long, with one mid-ventral seta. Segment 2 equilong to segment 1 with one mid-dorsal, one mid-ventral setae, and one long acerose on disto-ventral edge. Segment 3 very short and bluntly conical, with three distal strong long acerose setae. Limb with three simple endites, endites with two bare setae on tip, respectively. Epipod with one small bare inner seta and about seventeen long flexible setae.

**Seventh limb** (Figs. 3F, 6F): Limb simple, uniramous with two segments. Segment 1 slender and bare. Segment 2 very short and conical with two long flexible setae, the dorsal one about twice the length of another.

**Furca** (Figs. 3G, 6G): Each furcal lamella with one large ringed dorsal claw seta and seven claws with small ventral spines, arranged from long to short sequence. Furca without unpaired seta.

## Discussion

Initial examination identified these specimens in tribe Conchoeciini by features according to Chavtur and Angel’s key (Chavtur & Angel, 2011): (1) full adult size is in the range of 0.6 to 6.5 mm; (2) one asymmetric gland opens near postero-dorsal margin of left valve; (3) postero-dorsal corner is sharply angled; (4) segment 2 of first antenna carries a dorsal seta; (5) tip of 1st antenna bears five sensory setae; (6) endopod of second antenna has a processus mamillaris on anterior margin; (7) seventh limb is degraded; (8) each furca has one large seta and seven claws. Then they were tentatively identified as a species of genus *Conchoecissa* (Claus, 1890) by combined display of the characteristics (Chen & Lin, 1995; Angel, Blachowiak-Samolyk & Chavtur, 2008; Blachowiak-Samolyk & Angel, 2008): (1) shape of carapace is similar, posterior part of carapace is highest; (2) carapace surface is highly ornamented with polygonal pits; (3) rostrums are long and bent downward with pointed tip; (4) postero-dorsal corners have distinct but unequal spines; (5) postero-ventral corners are developed into tubercles; (6) setae of first antenna are homothetic; (7) endopod 1 of second antenna has a- and b-setae; (8) length of mandibular coxale is more than 1/4 of total length of mandible; (9) maxillary endopod 1 has six anterior setae; (10) caudal furcas are same, claws are long sickle shaped with numerous ventral small spines. They carapace outline is very similar to that of C. *plinthina.*

Detailed examination of the specimens showed they are not conspecific with any of the previously described *Conchoecissa* species (Müller, 1906; Chen & Lin, 1995; Chavtur, 2003; Chavtur & Angel, 2011; Drapun & Smith, 2012). For example, in comparisons between the specimens and the known *Conchoecissa* species (Table 1), it is obvious that the specimens are prominently bigger than known *Conchoecissa* species, and about 10% longer than C. *plinthina*, which is the biggest species of genus *Conchoecissa*, but the percentage of the 1st antenna to carapace is very much smaller in the novel species than in C. *plinthina* (about 49.0%: 71.4%, respectively). This novel species differs from the other *Conchoecissa* species in other several important characteristics (Table 2):

**Table 1:** Lengths of *Conchoecissa* species (♀) (MV Angel, 1935, 1972–1976, unpublished data). The data are expressed as percentages of the carapace length.

Species		*C. ametra*	*C. imbricata*	*C. plinthina*	*C. squamosa*	*C. symmetrica*	*C. nigromaculatus*
Carapace	Individuals	48	105	50	1	86	2
	Rang	3.48–4.40 mm	2.56–3.04 mm	4.92–5.83 mm	4.28 mm	4.00–4.72 mm	5.80–6.10 mm
	Length	100 (4.01 mm)	100 (2.77 mm)	100 (5.45 mm)	100 (4.28 mm)	100 (4.33 mm)	100 (5.95 mm)
	Height	41.7	40.0	nd	nd	nd	44.5
	Breadth	33.3	28.0	nd	nd	nd	32.8
	Left rostrum	10.4	25.9	nd	nd	nd	7.5
	Right rostrum	8.4	24.5	nd	nd	nd	6.9
Frontal organ	Stem	18.3	20.8	nd	nd	nd	16.7
	Capitulum	14.2	13.3	nd	nd	nd	17.7
1st antenna	Length (without setae)	14.4	15.2	nd	nd	nd	22.4
	Dorsal seta	13.0	8.2	nd	nd	nd	19.8
	A-d setae	10.6	10.7	nd	nd	nd	18.1
	E-seta	34.9	30.5	nd	nd	nd	51.8
2nd antenna	Exopod 1	13.7	11.4	nd	nd	nd	13.7
	G-seta	47.6	37.4	nd	nd	nd	34.6
6th limb	Longest terminal seta	12.3	11.4	nd	nd	nd	11.4
Furca	Longest terminal seta	14.3	11.7	nd	nd	nd	13.4

**Table 2:** Comparisons between *Conchoecissa nigromaculatus* sp. nov. and other species of *Conchoecissa* (♀).

Characteristics		*C. ametra* Müller, 1906	*C. imbricate* Brady, 1880	*C. plinthina* Müller, 1906	*C. squamosa* Müller, 1906	*C. symmetrica* Müller, 1906	C. *nigromaculatus*
Carapace	Rostrum	Short, base broad	Thin and long, base narrow	Long, base broad	Short, base narrow	Long, base narrow	Long, base broad
	Antero-ventral margins	None gland	None gland	None gland	None gland	None gland	A pair of edge glands
	Postero-ventral Corners	Armed	Armed	Slightly developed	Short and conical	Short and conical	Slightly developed
	Postero-dorsal Corners	Armed	Strongly Armed	Armed	Acutangular	Armed	Acutangular
E-seta of 1st antenna		Ventral spines	Ventral spines	Ventral spines	Ventral spines	Ventral spines	Ventral and dorsal spines
Mandibular coxal endite		With ventral finger process	With ventral finger process	With ventral finger process	With ventral finger process	With ventral finger process	Without ventral finger process
Terminal segment of Maxilla		Three claws	Three claws	Three claws	Three claws	Three claws	Two claws
6th limb	Endopod 3	Mid-dorsal and mid-ventral setae	Mid-dorsal and mid-ventral setae	Mid-dorsal and mid-ventral setae	Mid-dorsal and mid-ventral setae	Mid-dorsal and mid-ventral setae	Mid-dorsal seta, mid-ventral seta, and one long acerose disto- ventral seta
	Setae on tip	Three claws	Three claws	Three claws	Three claws	Three claws	Three long acerose setae
	Endites	Normal	Normal	Normal	Normal	Normal	Simple, with two bare setae on tip
Unpaired seta of Furca		Yes	Yes	Yes	Yes	Yes	None

(1) Each valve has one edge gland opening on antero-ventral margin, respectively.
(2) Postero-dorsal corners of the carapace are not-armed with spines. *C. squamosa* also lacks spines, but is obviously dissimilar.
(3) E-seta of first antenna is armed both dorsally and ventrally with fine spines, whereas the other *Conchoecissa* species have only ventral spines.
(4) Mandibular coxal endite has no ventral finger process.
(5) Endites of maxilla are larger than in other *Conchoecissa* species.
(6) Terminal segment of maxilla has only two claws; in other *Conchoecissa* species there are three.
(7) Basale of the sixth limb has a proximo-ventral seta.
(8) Endopod 2 of the sixth limb carries an additional long acerose seta on disto-ventral edge.
(9) Endites of the sixth limb are simple.
(10) There is no unpaired seta on the furcal lamellae.

It is noteworthy that many species of this tribe that have their gland locations shifted from basic locations are deep-living, usually associated with the secretion of bioluminescence. (Chavtur & Angel, 2011). The erection of this new species is based on carapace and locations of glands, as well as setae and structures of the limbs. However, Drapun proposed that some of the setal counts may well prove to be erroneous (Chavtur & Angel, 2011). Thus, the shape of carapace and locations of glands are just as significant as taxonomic characteristics of the tribe Conchoeciini (Müller, 1894; Angel & Iliffe, 1987). For example, the diagnosis of genus *Conchoecilla* (Claus, 1890) is indicated by one asymmetric gland located near postero-ventral margin of the left valve. Similarly, Poulsen (1973) identified genus *Gaussicia* as having one compound gland located on mid-ventral margin of the right valve. Moreover, Chavtur & Angel (2011) utilized the different locations of asymmetric gland on the dorsal margin to revise genus *Metaconchoecia* (Granata & Caporiacco, 1949) and erected another nine genera *Juryoeoia*, *Deeveyoecia*, *Vityazoecia*, *Muelleroecia*, *Nasoecia*, *Austrinoecia*, *Clausoecia*, *Kyrtoecia* and *Rotundecia*. To be consistent with these previous obsevations, we also used the features as the bases to separate the new taxon. Additional different features are observed on the first antenna, mandible, maxilla, the sixth limb and furca.

Remarkably, the mandibular coxal endite has no ventral finger process, maxilla has prominently big endites and has only two claws on tip, the sixth limb has very simple endites, all distinctive features not previously observed in tribe Conchoeciini before. It is therefore necessary to emend the diagnosis of this group.

Due to the rapid development of deep-sea science in the past 10 years, many uniquely deep-sea organisms have been discovered (Brandt et al., 2007; Rogers et al., 2012; Rogacheva, Gebruk & Alt, 2013; Rouse et al., 2016; Kou, Gong & Li, 2018). However, our understanding of deep-sea organisms is still greatly inadequate, and a large number of deep-sea organisms are still unknown. The bathypelagic halocyprid specimens in this study were collected from the southern South China Sea in a region close to an ocean trench and an adjoining coral reef marine biodiversity hotspot. This kind of region represents a large reservoir of biomass with a large proportion of undiscovered biodiversity, (Roberts et al., 2002; Gianni, 2004; Danovaro et al., 2008). Our examinations of these specimens confirmed a novel bathypelagic halocyprid species, which increases the number of the planktonic ostracod species in this area. We can expect that there is also a highly diverse ostracod fauna in this region.

## Conclusions

The family Halocyprididae is a diverse group of ostracods. In this study, we described a new bathypelagic species *C. nigromaculatus* that differed from the other halocyprid ostracods in having the combination of characteristics of size, glands, e-seta of the first antenna, coxal endite of mandible, endites of maxilla, setae and structure of the sixth limb, and unpaired seta of furca. The mandibular coxal endite has no ventral finger process; maxilla has prominently big endites and the sixth limb has very simple endites; have distinctive features not previously observed in tribe Conchoeciini before. Our examinations of the specimens confirmed a novel bathypelagic halocyprid species, which increases the records of planktonic ostracods in the central and southern part of the South China Sea.

Finally, more detailed observations of these species are needed in order to update the diagnosis of this group, and more intensive studies are needed in order to reveal cryptic diversity of ostracods in the relatively underexplored abyssal South China Sea.

##  Supplemental Information

10.7717/peerj.5557/supp-1 Table S1 Checklist and seasonal distribution of planktonic ostracods in the central and southern part of the South China Sea

## Additional Information and Declarations

### Data Availability

The following information was supplied regarding data availability: Specimens/appendages have been archived in the Marine Biological Sample Museum, the Third Institute of Oceanography, SOA (Xiamen, China), under the collection Nos. TIO-OHHZe-01 and TIO-OHHZe-02.

### New Species Registration

The following information was supplied regarding the registration of a newly described species: Publication LSID: urn:lsid:zoobank.org:pub:D5DFB0CC-37C5-4A75-BB4B-5A4FF9A81747 Species: urn:lsid:zoobank.org:act:AE68FC48-CBD1-4090-84AE-27913DB59BA5.
