# Supplementary material for: A bathypelagic ostracod Conchoecissa nigromaculatus sp. nov. (Myodocopa, Halocyprididae) from the South China Sea
Source: PeerJ. 2018 Sep 7;6:e5557. doi: 10.7717/peerj.5557 (PMC6130238; doi:10.7717/peerj.5557)
Supplement: Table S1 [file peerj-06-5557-s001.docx]

Table S1 Checklist and seasonal distribution of planktonic ostracods in the central and southern part of the South China Sea

| Family | Subfamily | Tribe | Species | Reference | Spring | Summer | Autumn | Winter |
| --- | --- | --- | --- | --- | --- | --- | --- | --- |
| Cylindroleberididae | Cyclasteropinae |  | *Tetraleberis brevis* | Brady, 1902 |  | + |  |  |
| Cypridinidae | Cypridininae | Gigantocypridinini | *Gigantocypris agassizi* | Müller, 1895 |  |  |  | + |
|  |  |  | *Gigantocypris australis* | Poulsen, 1962 |  | + | + |  |
|  |  | Cypridinini | *Cypridina acuminata* | Müller, 1906 | + |  |  | + |
|  |  |  | *C. dentata* | Müller, 1906 | + |  |  | + |
|  |  |  | *C. flaveola* | Claus, 1873 |  | + |  |  |
|  |  |  | *C. nana* | Poulsen, 1962 |  |  | + |  |
|  |  |  | *C. serrata* | Müller, 1906 | + |  | + | + |
|  |  |  | *Paravargula hirsuta* | Müller, 1906 | + |  |  | + |
|  |  |  | *Melavargula japonica* | Poulsen, 1962 | + |  | + |  |
| Halocyprididae | Archiconchoeciinae |  | *Archiconchoecia falcata* | Deevey, 1978 | + |  |  |  |
|  | Bathyconchoeciinae |  | *Bathyconchoecia incisa* | Yin, et al., 2017 |  | + |  |  |
|  |  |  | *B. lacunosa* | Müller, 1908 |  |  | + |  |
|  |  |  | *B. liui* | Yin, et al., 2014 |  | + |  |  |
|  | Conchoeciinae | Conchoeciini | *Alacia belgicae* | Müller, 1906 |  |  | + |  |
|  |  |  | *A. hettacro* | Müller, 1906 | + |  |  |  |
|  |  |  | *A. leptothrix* | Müller, 1906 |  | + |  |  |
|  |  |  | *A. valdiviae* | Müller, 1906 |  |  | + |  |
|  |  |  | *Conchoecetta acuminata* | Claus, 1890 |  | + | + |  |
|  |  |  | *C. giesbrechti* | Müller, 1906 |  | + |  |  |
|  |  |  | *Conchoecia hyalophyllum* | Claus, 1890 |  | + |  |  |
|  |  |  | *C. lophura* | Müller, 1906 |  |  |  |  |
|  |  |  | *C. macrocheira* | Müller, 1906 |  | + | + | + |
|  |  |  | *C. magna* | Claus, 1874 | + | + | + | + |
|  |  |  | *C. magna rhombica* | Müller, 1906 | + | + |  |  |
|  |  |  | *C. parvidentata* | Müller, 1906 | + |  |  | + |
|  |  |  | *C. subarcuata* | Claus, 1890 |  | + |  |  |
|  |  |  | *Conchoecilla daphnoides* | Claus, 1890 |  |  | + | + |
|  |  |  | *Conchoecissa ametra* | Müller, 1906 | + |  |  | + |
|  |  |  | *C. imbricata* | Brady, 1880 |  | + |  |  |
|  |  |  | *C. nigromaculatus* | Xiang, et al., Herein |  |  |  | + |
|  |  |  | *C. plinthina* | Müller, 1906 |  |  | + |  |
|  |  |  | *C. symmetrica* | Müller, 1906 |  |  |  | + |
|  |  |  | *Discoconchoecia elegans* | Sars, 1866 | + | + | + |  |
|  |  |  | *D. pseudodiscophora* | Rudjakov, 1962 |  |  | + |  |
|  |  |  | *D. tamensis* | Poulsen, 1973 |  | + |  |  |
|  |  |  | *Gaussicia incisa* | Müller, 1906 |  |  | + |  |
|  |  |  | *Loricoecia loricata* | Claus, 1894 |  |  | + |  |
|  |  |  | *L. ctenophora* | Müller, 1906 |  |  | + |  |
|  |  |  | *Mikroconchoecia curta* | Lubbock, 1860 | + | + |  | + |
|  |  |  | *Orthoconchoecia bispinosa* | Claus, 1890 | + | + |  |  |
|  |  |  | *O. striola* | Müller, 1906 | + |  | + |  |
|  |  |  | *O. atlantica* | Lubbock, 1856 | + | + |  | + |
|  |  |  | *O. secernenda* | Vávra, 1906 |  |  |  | + |
|  |  |  | *Paraconchoecia aequiseta* | Müller, 1906 |  | + |  |  |
|  |  |  | *P. allotherium* | Müller, 1906 |  | + |  |  |
|  |  |  | *P. brachyaskos* | Müller, 1906 |  | + | + |  |
|  |  |  | *P. cophopyga* | Müller, 1906 |  | + |  |  |
|  |  |  | *P. dasyophthalma* | Müller, 1906 |  |  | + |  |
|  |  |  | *P. decipiens* | Müller, 1906 |  | + | + |  |
|  |  |  | *P. dentata* | Müller, 1906 |  |  |  | + |
|  |  |  | *P. echinata* | Müller, 1906 |  | + |  |  |
|  |  |  | *P. inermis* | Claus, 1890 |  | + |  |  |
|  |  |  | *P. mamillata* | Müller, 1906 |  |  | + |  |
|  |  |  | *P. macroprocera* | Angel, 1971 |  | + |  |  |
|  |  |  | *P. microprocera* | Angel, 1971 |  | + |  |  |
|  |  |  | *P. oblonga* | Claus, 1891 | + | + | + | + |
|  |  |  | *P. procera* | Müller, 1894 | + | + | + | + |
|  |  |  | *P. reticulata* | Müller, 1906 |  |  | + |  |
|  |  |  | *P. spinifera* | Claus, 1890 |  | + |  |  |
|  |  |  | *P. vitjazi* | Rudjakov, 1962 |  | + |  |  |
|  |  |  | *Paramollicia plactolycos* | Müller, 1906 | + |  |  |  |
|  |  |  | *Porroecia crassispina* | Chen & Lin, 1987 | + | + |  |  |
|  |  |  | *P. parthenoda* | Müller, 1906 | + | + |  | + |
|  |  |  | *P. porrecta* | Claus, 1890 | + | + | + | + |
|  |  |  | *P. pseudoparthenoda* | Angel, 1972 |  | + |  |  |
|  |  |  | *P. spinirostris* | Claus, 1874 |  | + |  | + |
|  |  |  | *Pseudoconchoecia concentrica* | Müller, 1906 | + | + |  | + |
|  |  | Metaconchoeciini | *Metaconchoecia abyssalis* | Rudjakov, 1962 |  |  | + |  |
|  |  |  | *M. Clausoecia* | Müller, 1906 | + |  |  |  |
|  | Euconchoeciinae |  | *Euconchoecia aculeata* | Scott, 1894 | + |  |  | + |
|  |  |  | *E. bifurata* | Chen & Lin, 1984 | + |  |  |  |
|  |  |  | *E. elongata* | Müller, 1906 | + |  |  | + |
|  |  |  | *E. maimai* | Tseng, 1969 | + |  |  | + |
|  | Halocypridinae |  | *Fellia bicornis* | Müller, 1906 |  |  | + |  |
|  |  |  | *Halocypria globosa* | Claus, 1874 | + |  |  |  |
|  |  |  | *Halocypris inflata* | Dana, 1849 | + | + | + | + |
|  |  |  | *Muelleroecia macromma* | Müller, 1906 |  |  |  |  |
| Philomedidae | Philomedinae |  | *E. biacutidens* | Xiang, et al., 2017 |  | + |  |  |
|  |  |  | *Philomedes eugeniae* | Skogsberg, 1920 |  |  | + |  |
|  |  |  | *Euphilomedes interpuncta* | Baird, 1850 | + |  |  |  |

**References**

1. **Angel MV. 1971.** *Conchoecia from the North Atlantic: The" procera" Group.* British Museum (Natural History) Zoology.
2. **Angel MV. 1972.** *Conchoecia Pseudoparthenoda (nov. Sp): A New Halocyprid Ostracod for the Tropical North Atlantic*. British Museum.
3. **Baird W. 1850.** Description of several new species of Entomostraca. *Proc. Zool. Soc. Lond.* 18:254-257.
4. **Brady GS. 1880.** Report on the Ostracoda dredged by HMS Challenger during the years 1873-1876. *Zoology* 1:1-184.
5. **Brady GS. 1902.** On new or imperfectly known Ostracoda, chiefly from a collection in the Zoological Museum, Copenhagen. *Transactions of the Zoological Society of London* 16(4):179–210.
6. **Chen R, Lin J. 1984.** A new species of Euconchoecia from the East China Sea. *Acta Oceanologica Sinica* 5:859-861.
7. **Chen R, Lin J. 1987.** Spinoecia crassispina (nov. sp.), a new species of planktonic Ostracoda. *Acta Oceanologica Sinica* 1:017.
8. **Claus C. 1873.** Neue Beobachtungen ueber Cypridinen. *Zeitschrift fuer wissenschaftliche Zoologie* 23:211–227.
9. **Claus C. 1874.** Die Gattungen und Arten der Halocypriden. V*erh. Zool. Bot. Ges. Wien* 24:175-178.
10. **Claus C.** **1874.** Die Gattungen und Arten der Halocypriden. *Verh. Zool. Bot. Ges. Wien* 24:175-178.
11. **Claus C. 1890.** Die Gattungen und Arten der mediterranen und atlan tischen Holocypriden. *Zool Inst Univ Wien Arb* 9(1):1-34.
12. **Claus C. 1891.** Ueber das Verhalten des nervosen Endapparates an den Sinneshaaren der Crustaceen. *Zool. Anz* 14:363-368.
13. **Claus C. 1894.** *Die Halocypriden und ihre Entwicklungsstadien: gesammelt 1890, 1891, 1892, 1893.* Kaiserlich-Königliche Hof-und Staatsdr.
14. **Dana JD. 1849.** *United States Exploring Expedition During the Years 1838, 1839, 1840, 1841, 1842 Under the Command of Charles Wilkes, USN: 10: Geology*. Printed by C. Sherman.
15. **Deevey GB. 1978.** The planktonic ostracods of the Cariaco Trench and adjacent waters. *Proceedings of the Biological Society of Washington* 91(1):53–73.
16. **Lubbock J. 1856.** II. On some Entomostraca collected by Dr. Sutherland, in the Atlantic Ocean. *Transactions of the Royal Entomological Society of London* 9(2):8-37.
17. **Lubbock J. 1860.** XV. On some Oceanic Entomostraca collected by Captain Toynbee. *Transactions of the Linnean Society of London* 23(1):173-191.
18. **Müller GW. 1894.** Die Ostracoden des Golfes von Neapel und der angrenzenden Meeres-Abschnitte. In: Fauna und Flora des Golfes von Neapel und der Angrenzenden Meeres-Abschnitte. *Herausgegeben von der Zoologischen Station zu Neapel* 21:1-404.
19. **Müller GW. 1895.** Reports on the dreging operations off the west coast of central America to the Galapagos to the west coast of Mexico, and in the Gulf of California, in charge of Alexander Agassiz, carried on by the U.S. Fish Commission streamer “Albatross”, during 1891. *In: U. S. F. C. Marshall McDonald (Ed), Bulletin of the Museum of Comparative Zoology at Harvard College.* Printed for the Museum, Cambridge, Mass., U.S.A. 155–169.
20. **Müller GW. 1906.** Ostracoda. *Wiss Erg `Valdivia'* 8:27–154.
21. **Müller GW. 1908.** Die Ostracoden der Deutschen Südpolar-Expedition 1901-1903. *Deutschen Südpolar Expedition* 10:50-181.
22. **Poulsen EM. 1962.** Ostracoda-Myodocopa, Part I: Cyridiniformes-Cypridinidae. *Dana Report* 57:1–414.
23. **Poulsen EM. 1973.** Ostracoda – Myodocopa, Part III B, Halocypriformes – Halocypridae Conchoecina. *Dana Repart* 84:1-223.
24. **Rudjakov JA.** 1962. Ostracoda of the shallows of the Kandalaksha (White Sea). *Trudy Belom. Biol. Sta. MGU* 1:130-142.
25. **Sars GO. 1866.** Oversigt af Norges marine Ostracoder. *Forhandlinger i Videnskabs-Selskabet i Christiania* 7:1-130.
26. **Scott T. 1894.** I. Report on Entomostraca from the Gulf of Guinea, collected by John Rattray, B. Sc. *Transactions of the Linnean Society of London. 2nd Series: Zoology* 6(1):1-161.
27. **Skogsberg T.** *1920. Studies on marine ostracods Vol. 15.* Almqvist & Wiksells.
28. **Tseng W. 1969.** *Euconchoecia* (Ostracoda) from Taiwan Straits. *Reports of the Taiwan Laboratory of Fish Biology* 19:1-26.
29. **Vávra, V. 1906.** *Die Ostracoden (Halocypriden und Cypridiniden) der Plankton-Expcditio. E,'gebn. Plankton-Exped. II.* Kielnnd Leipzig.
30. **Xiang P, Ye Y, Chen X, Chen R, Lin M. 2017.** *Euphilomedes biacutidens* (Ostracoda, Myodocopida, Philomedidae), a new species from China Sea. *PeerJ* 5:e3488.
31. **Yin J, Chen Q, Li K. 2014.** *Bathyconchoecia liui* n. sp., a new species of ostracod (Myodocopa, Halocyprididae) from the South China Sea. *Crustaceana* 87(8-9):1027-1035.
32. **Yin J, Li K, Tan Y. 2017.** *Bathyconchoecia incisa* sp. nov. (Myodocopa, Halocyprididae), a new species of ostracod from the neritic zone of the South China Sea. *Crustaceana* 90(1):35-48
